# Supplementary material for: Serotonin reuptake inhibitors improve muscle stem cell function and muscle regeneration in male mice
Source: Nat Commun. 2024 Jul 31;15:6457. doi: 10.1038/s41467-024-50220-4 (PMC11291725; doi:10.1038/s41467-024-50220-4)
Supplement: Supplementary file 1 — Supplementary Information [file 41467_2024_50220_MOESM1_ESM.pdf]

SUPPLEMENTARY INFORMATION FILE

Serotonin reuptake inhibitors improve muscle stem cell function and muscle regeneration in male mice

Mylène Fefeu[1,2,3], Michael Blatzer[2], Anita Kneppers[4], David Briand[2], Pierre Rocheteau[2], Alexandre Haroche[1], David Hardy[2], Mélanie Juchet-Martin[2], Anne Danckaert[5], François Coudoré[6], Abdulkarim Tutakhail[6], Corinne Huchet[7], Aude Lafoux[8], Rémi Mounier[4], Olivier Mir[9], Raphaël Gaillard[1,2,3], Fabrice Chrétien[2,3,10].

1: GHU Paris Psychiatrie & Neurosciences, site Sainte Anne, Service Hospitalo-Universitaire de psychiatrie, Paris, France
2: Institut Pasteur, Experimental Neuropathology Unit, Global Health Department, Paris, France
3: Université de Paris Cité, Paris France
4: Institut NeuroMyoGène, Unité Physiopathologie et Génétique du Neurone et du Muscle, Université Claude Bernard Lyon 1, CNRS UMR 5261, Inserm U1315, Univ Lyon, Lyon, France
5: Institut Pasteur, UTechS PBI, C2RT, Paris France
6: CESP, MOODS Team, Inserm, Faculté de Pharmacie, Université Paris-Saclay, Châtenay-Malabry, France
7: TaRGeT, INSERM UMR 1089, Nantes Université, CHU Nantes, Nantes, France
8: Therassay Platform, Capacités, Université de Nantes, IRS 2 Nantes Biotech, Nantes, France
9: Sarcoma Group, Gustave Roussy, Villejuif, France
10 : GHU Paris Psychiatrie & Neurosciences, site Sainte Anne, Service Hospitalo-Universitaire de neuropathologie, Paris, France

Corresponding authors: Pr. Fabrice Chrétien & Pr Raphaël Gaillard, GHU Paris Psychiatrie & Neurosciences, 1 rue Cabanis, 75014 Paris, France. Tel +33-145658261
f.chretien@ghu-paris.fr ; raphael.gaillard@normalesup.org
These authors contributed equally: Anita Kneppers, David Briand, Pierre Rocheteau.
These authors jointly supervised this work: Raphaël Gaillard, Fabrice Chrétien.

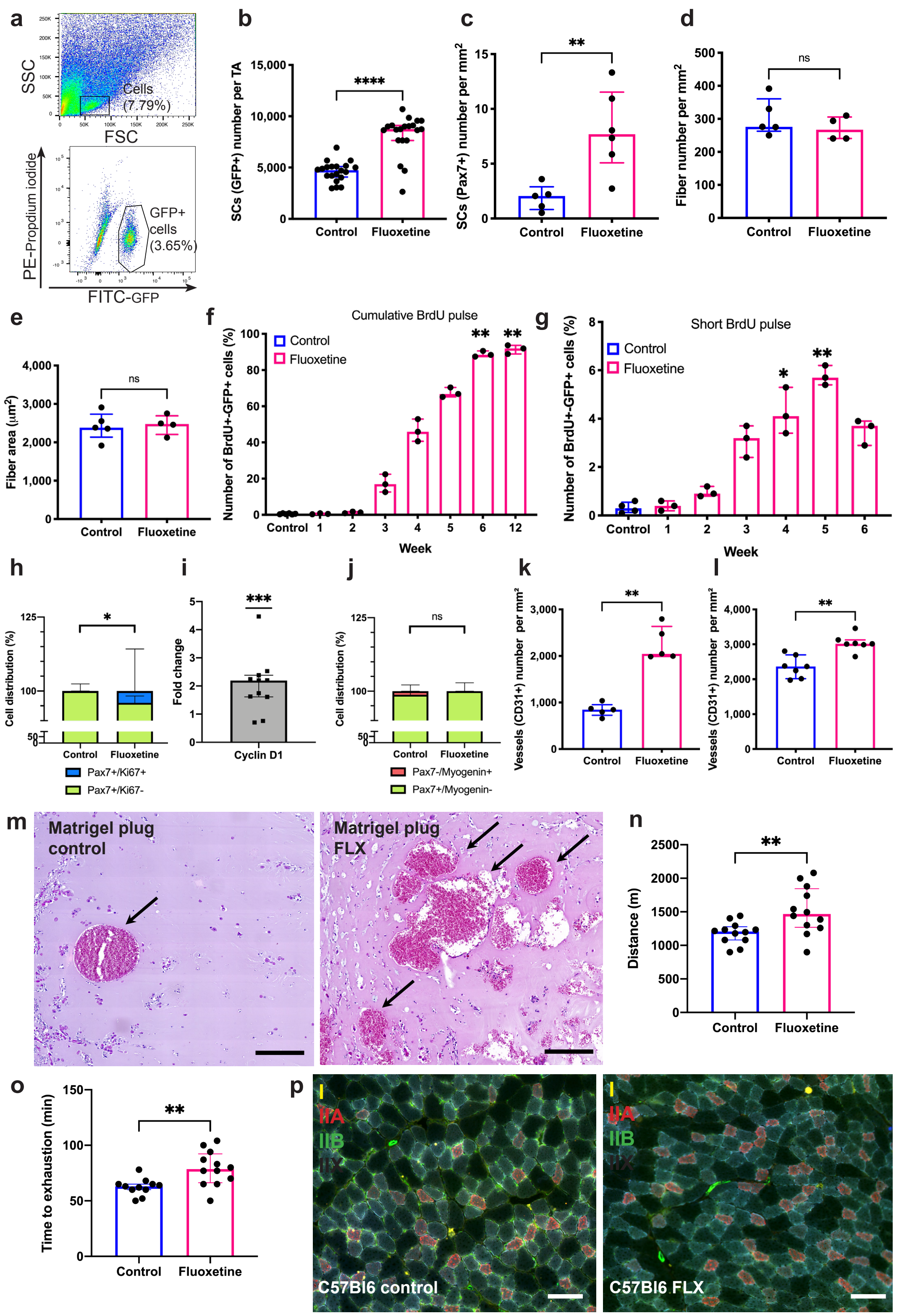

Supplementary Figure 1: Fluoxetine increases satellite cell and vessel numbers with physiologic and metabolic changes in skeletal muscle at steady state.

(a) Cytometric representation of SCs-GFP+ of TA from *Tg:Pax7nGFP* control mice. **(b)** Quantification of the SCs (GFP+) number per TA from *Tg:Pax7nGFP* mice treated with fluoxetine (FLX) or control with FACS analysis (n=20 per condition, 3 independent experiments). **(c)** Quantification of the SCs (Pax7+) number on TA sections from control and FLX-treated C57Bl6 mice (n=5 control, 6 FLX). **(d)** Quantification of the muscle fibers number on TA sections from control and FLX-treated C57Bl6 mice (n=5 control, 4 FLX). **(e)** Area of muscle fibers on TA sections from control and FLX-treated C57Bl6 mice (n=5 control, 4 FLX). **(f)** Percentage of cumulative divided SCs (BrdU+) after FACS-sorted GFP+ cells from control and FLX-treated *Tg:Pax7nGFP* mice (n= 6 control, 3 FLX per time point). **(g)** Percentage of dividing SCs (BrdU+) after FACS-sorted GFP+ cells from control and FLX-treated *Tg:Pax7nGFP* mice (n= 4 control, 3 FLX per time point). **(h)** Cell distribution (%) of Pax7+ and Ki67+ among SCs on TA sections from control and FLX-treated C57Bl6 mice (n=5 per condition). **(i)** mRNA expression level of *Cyclin D1* gene by RT-qPCR in FACS-sorted SCs from control and FLX-treated *Tg:Pax7nGFP* mice (n=7 control, 11 FLX; 2 independent experiments). **(j)** Cell distribution (%) of Pax7+ and myogenin+ among SCs on TA sections from control and FLX-treated C57Bl6 mice (n=7 per condition). **(k)** Quantification of the vessel (CD31+) numbers on TA sections from control and FLX-treated C57Bl6 mice (n=5 per condition). **(l)** Quantification of the vessel (CD31+) numbers on Soleus sections from control and FLX-treated C57Bl6 mice (n=7 per condition). **(m)** Representative Hematoxylin and Eosin-stained cross sections of Matrigel® plugs from control and FLX-treated recipient mice. Arrows indicated functional vessels containing red blood cells). **(n)** Distance traveled determined by treadmill exercise of FLX-treated and control C57Bl6 mice (n=12 per condition; 2 independent experiments). **(o)** Time to exhaustion determined by treadmill exercise of FLX-treated and control C57Bl6 mice (n=11 control, 12 FLX; 2 independent experiments). **(p)** Representative histological TA sections of control and FLX-treated C57Bl6 mice; sections display MyHC IIB (green), MyHC IIA (red), MyHC I (yellow), MyHC IIX (black). Scale bars indicate 100 µm. All values are represented as median with interquartile range. The two-tailed Mann-Whitney test for **(b)-(e), (h), (j)-(l), (n), (o)**, the

Kruskal-Wallis test for **(f), (e)** and the Wilcoxon test for **(i)**. * p≤0.05, ** p≤0.01, ***p≤0.001, ****p≤0.0001. Source data are provided as a Source Data file.

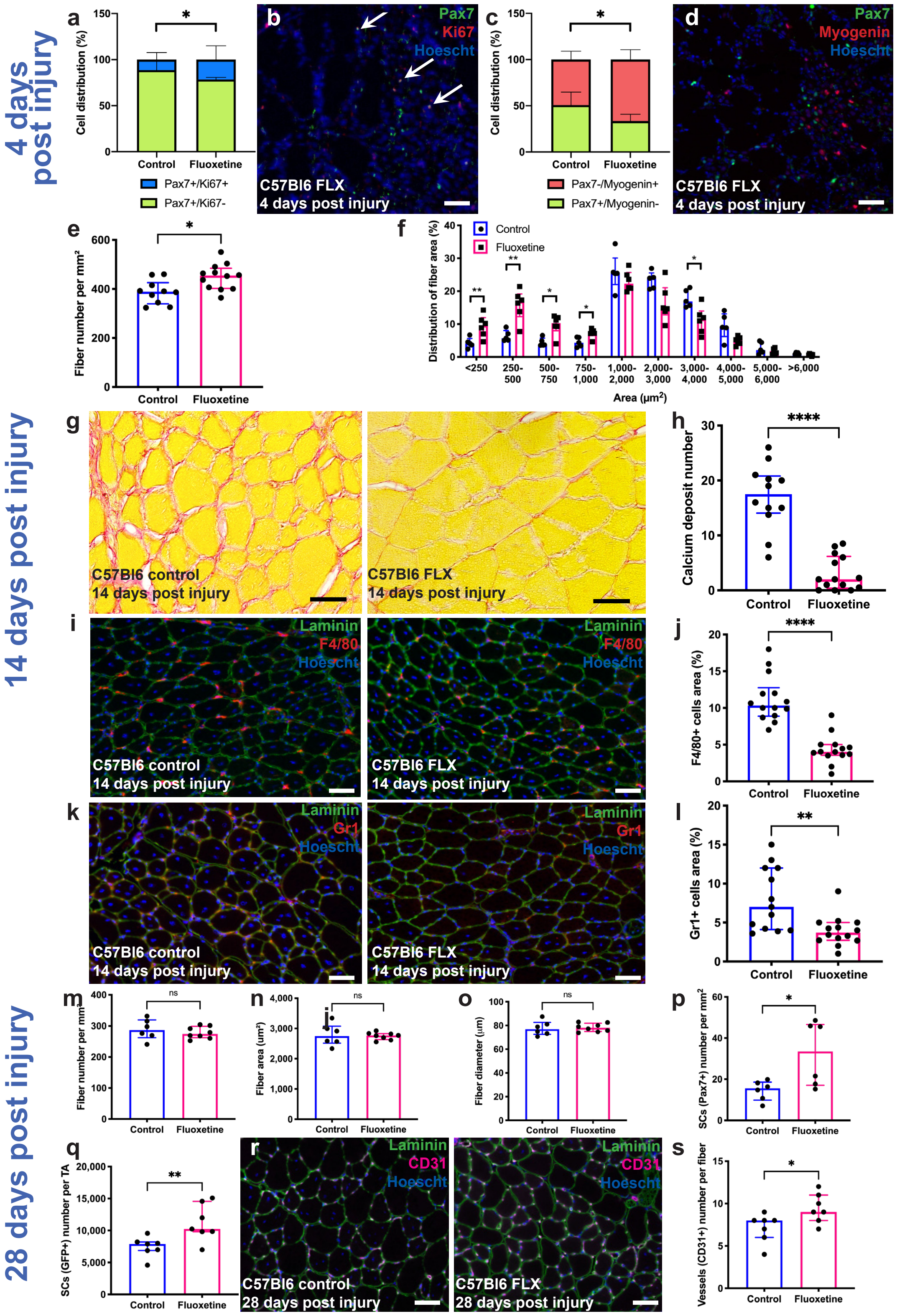

Supplementary Figure 2: Fluoxetine accelerates muscle regeneration *ad integrum* after injury.

(a) Cell distribution (%) of Pax7+ and Ki67+ among SCs 4 days post injury on TA sections from control and fluoxetine (FLX)-treated C57Bl6 mice (n=7 per condition). **(b)** Representative immunostaining TA sections of FLX-treated mice 4 days post injury; sections display Pax7 (green), Ki67 (red), Hoechst (nuclei, blue), the arrows indicate the Pax7+/Ki67+ cells. **(c)** Cell distribution (%) of Pax7+ and myogenin+ among SCs 4 days post injury on TA sections from control and FLX-treated C57Bl6 mice (n=6 control, 7 FLX). **(d)** Representative immunostaining TA sections of FLX-treated mice 4 days post injury; sections display Pax7 (green), myogenin (red), Hoechst (nuclei, blue). **(e)** Quantification of the fibers number 14 days post injury on TA sections from control and FLX-treated C57Bl6 mice (n=10 control, 12 FLX; 2 independent experiments). **(f)** Distribution of fiber area 14 days post injury on TA sections from control and FLX-treated C57Bl6 mice (n=5 control, 6 FLX). **(g)** Representative Sirius Red-stained TA sections of control and FLX-treated mice 14 days post injury. **(h)** Quantification of the calcium deposit number 14 days post injury on TA sections from control and FLX-treated *Tg:Pax7nGFP* mice (n=12 control, 14 FLX; 2 independent experiments). **(i)** Representative immunostaining TA sections of control and FLX-treated mice 14 days post injury; sections display Laminin (green), F4/80 (macrophages; red), Hoechst (nuclei, blue). **(j)** Percentage of F4/80+ immune cells infiltration areas 14 days post injury on TA sections from control and FLX-treated *Tg:Pax7nGFP* mice (n=14 per condition; 2 independent experiments). **(k)** Representative immunostaining TA sections of control and FLX-treated mice 14 days post injury; sections display Laminin (green), Gr1 (granulocyes; red), Hoechst (nuclei, blue). **(l)** Percentage of Gr1+ immune cells infiltration areas 14 days post injury on TA sections from control and FLX-treated *Tg:Pax7nGFP* mice (n=13 control, 14 FLX; 2 independent experiments). **(m)** Quantification of the fibers number 28 days post injury on TA sections from control and FLX-treated C57Bl6 mice (n=6 control, 8 FLX). **(n)** Area of muscle fibers 28 days post injury on TA sections from control and FLX-treated C57Bl6 mice (n=6 control, 8 FLX). **(o)** Quantification of muscle fiber diameter 28 days post injury on TA sections from control and FLX-treated C57Bl6 mice (n=6 control, 8 FLX). **(p)** Quantification of the SCs (Pax7+) number 28 days post injury on TA sections from control and FLX-treated

C57Bl6 mice (n=6 per condition). **(q)** Quantification of the SCs (GFP+) number per TA with cytometry analysis 28 days post injury from *Tg:Pax7nGFP* control or FLX-treated mice (n=7 per condition). **(r)** Representative immunostaining TA sections of control and FLX-treated mice 28 days post injury; sections display Laminin (green), CD31 (pink), Hoechst (nuclei, blue). **(s)** Quantification of the vessel (CD31+) numbers per muscle fiber on TA sections from control and FLX-treated C57Bl6 mice (n=7 mice per condition). All scale bars indicate 50 µm. All values are represented as median with interquartile range. All data analyses were performed with the two-tailed Mann-Whitney test. * $p \le 0.05$, ** $p \le 0.01$, ****$p \le 0.0001$. Source data are provided as a Source Data file.

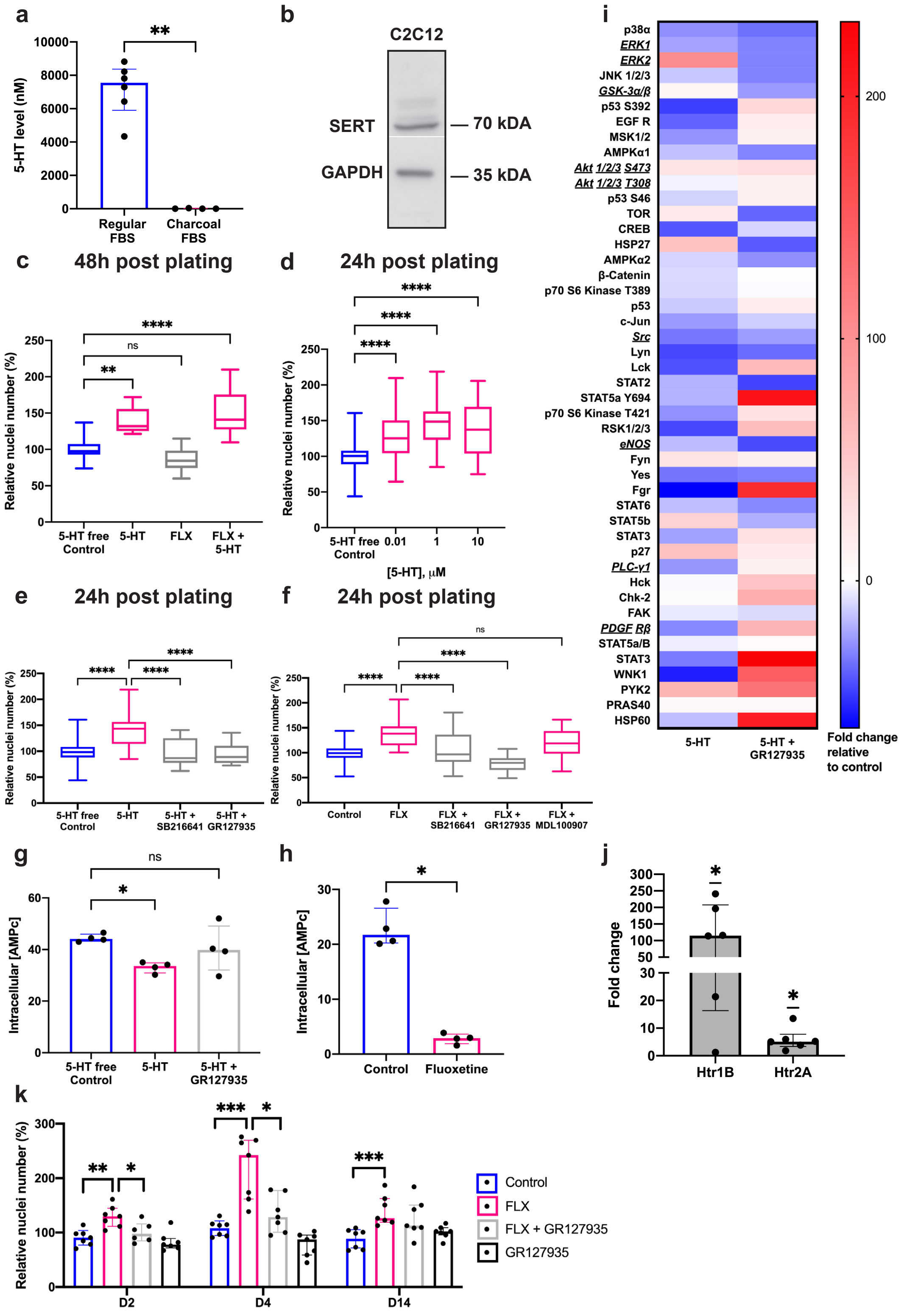

Supplementary Figure 3: Fluoxetine acts on myoblasts via the 5-HT1B receptor

(a) 5-HT levels in fetal bovine serum (FBS) and charcoal FBS assessed by HPLC (n=4

charcoal FBS, 6 FBS). **(b)** Representative western blot of the expressions of the targeted SERT protein and the housekeeping GAPDH protein in C2C12. **(c)** Percentage of the nuclei number in C2C12 cells after exposure to serotonin (5-HT), fluoxetine (FLX) and FLX + 5-HT in 5-HT-free medium for 2 days (3 independent experiments). **(d)** Percentage of the nuclei number in C2C12 cells after exposure to increasing doses of 5-HT for 24h in 5-HT-free medium (3 independent experiments). **(e)** Percentage of the nuclei number in C2C12 cells pretreated with specific antagonists (5-HT1B antagonists: SB216641 and GR127935) and exposed to 5-HT for 24h in 5-HT-free medium (3 independent experiments). **(f)** Percentage of the nuclei number in C2C12 cells pretreated with specific antagonists (5-HT1B antagonists: SB216641 and GR127935; 5-HT2A antagonist: MDL100907) and exposed to FLX for 24h in regular medium (3 independent experiments). **(g)** Intracellular cAMP levels in C2C12 after 30min exposure to 5-HT and 5-HT + GR127935 in 5-HT-free medium (4 independent experiments). **(h)** Intracellular cAMP levels in C2C12 after 30min exposure to FLX in regular medium (4 independent experiments). **(i)** Heat map of relative changes in protein kinases phosphorylation in C2C12 after 30min exposure to 5-HT and 5-HT + GR127935 in 5-HT-free medium (3 independent experiments). The underlined protein kinases correspond to the 5-HT1B receptor target proteins already identified in other cell lines [20]. **(j)** mRNA expression level of Htr1B and Htr2A genes by RT-qPCR in FACS-sorted SCs from control and FLX-treated *Tg:Pax7nGFP* mice (n=5 per condition). **(k)** Quantification of the relative number of nuclei (%) over time in FACS-sorted SCs from *Tg:Pax7nGFP* mice after exposure to FLX, FLX + GR127935, or GR127935, at 2 (D2), 4 (D4), and 14 (D14) days *post* plating in regular medium (n= 7 per condition). All values are represented as median with interquartile range. The two-tailed Mann-Whitney test for **(a), (h), (i)**, the Kruskal-Wallis test for **(c)-(g), (k)** and the Wilcoxon test for **(j)**. * $p \leq 0.05$, ** $p \leq 0.01$, **** $p \leq 0.0001$. Source data are provided as a Source Data file.

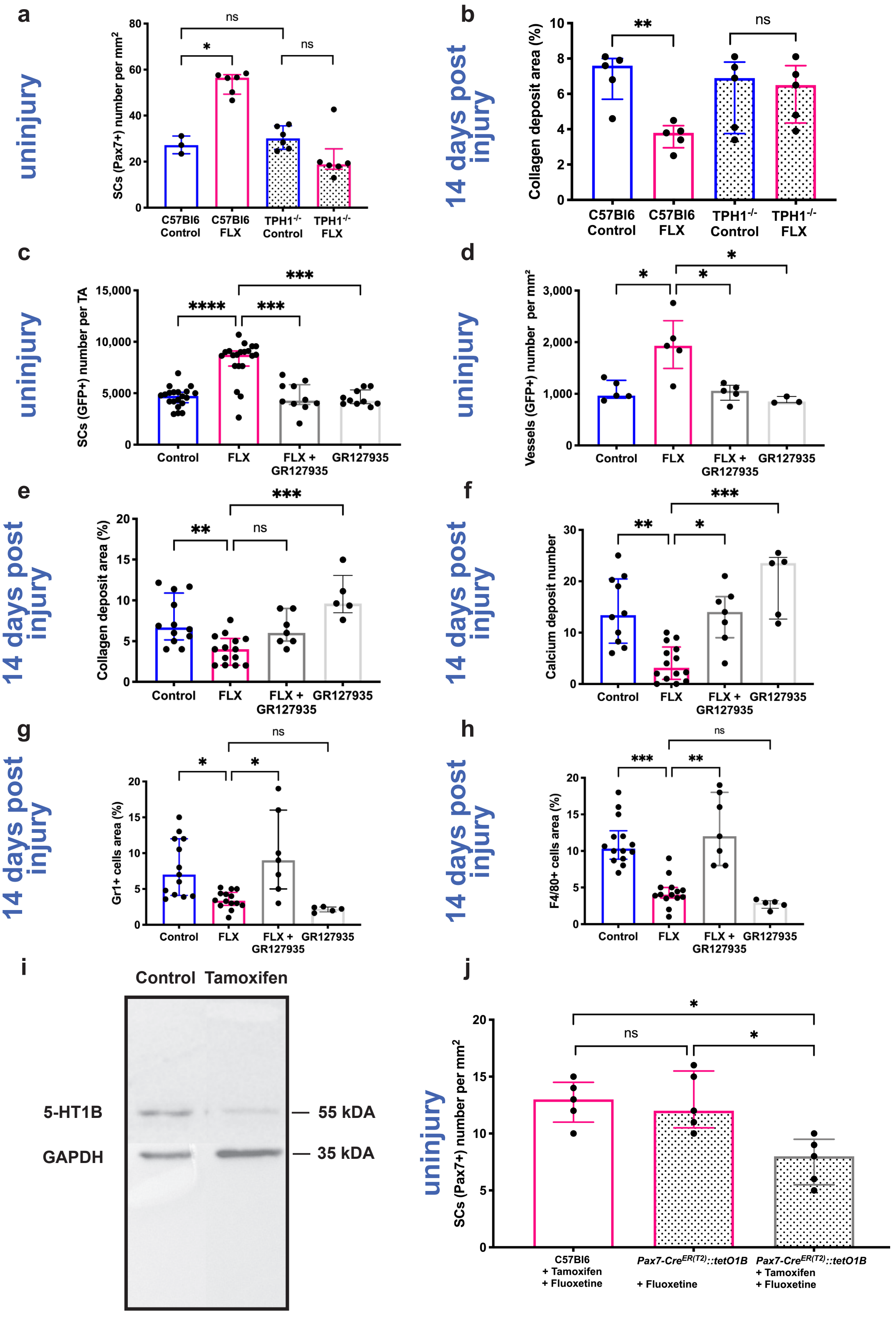

Supplementary Figure 4: Fluoxetine acts on muscle stem cells and muscle regeneration in a 5-HT and 5-HT1B dependent manner.

(a) Quantification of the SCs number by Pax7 immunostaining on TA sections from control and fluoxetine (FLX)-treated C57Bl6 and *TPH1*$^{-/-}$ mice (n=3 C57Bl6 control, 6 per other condition). **(b)** Percentage of the collagen deposit area stained with Sirius Red 14 days post injury on TA sections from control and FLX-treated C57Bl6 and *TPH1*$^{-/-}$ mice (n=5 per condition). **(c)** Quantification of the SCs (GFP+) number per TA with cytometry analysis from *Tg:Pax7nGFP* mice in FLX, FLX + GR127935, GR127935 and control group (n=20 control and FLX, 10 FLX + GR127935 and GR127935; 2 independent experiments). **(d)** Quantification of the vessels number by GFP immunostaining on TA sections from *Flk1*$^{GFP/+}$ mice in FLX, FLX + GR127935, GR127935 and control group (n=3 GR127935, 5 per other condition). **(e)** Percentage of collagen deposit area stained with Sirius Red 14 days post injury on TA sections from control, FLX, GR127935 and FLX+ GR127935-treated C57Bl6 mice (n=12 control, 14 FLX, 7 FLX+ GR127935, 5 GR127935). **(f)** Quantification of the calcium deposit number stained with Hematoxylin and Eosin 14 days post injury on TA sections from control, FLX, GR127935 and FLX+ GR127935-treated C57Bl6 mice (n=10 control, 14 FLX, 7 FLX+ GR127935, 5 GR127935). **(g)** Percentage of Gr1+ (granulocytes) and **(h)** F4/80+ (macrophages) immune cells infiltration areas 14 days post injury on TA sections from control, FLX, GR127935 and FLX+ GR127935-treated C57Bl6 mice (n=14 control, 14 FLX, 7 FLX+ GR127935, 5 GR127935). **(i)** Representative western blot of the expressions of the targeted 5-HT1B receptor protein and the housekeeping GAPDH protein in murine primary myoblasts isolated by pre-platting from *Pax7-Cre*$^{ER(T2)}$*::tetO1B* mice receiving the tamoxifen or the control (n=3-6 mice per condition). **(j)** Quantification of the SCs number by Pax7 immunostaining on TA sections from C57Bl6 and *Pax7-Cre*$^{ER(T2)}$*::tetO1B* mice receiving the control, tamoxifen and/or FLX (n=5 per condition). All values are represented as median with interquartile range. The Kruskal-Wallis test for **(a)-(h), (j).** * $p \leq 0.05$, ** $p \leq 0.01$, *** $p \leq 0.001$, ****$p \leq 0.0001$. Source data are provided as a Source Data file.

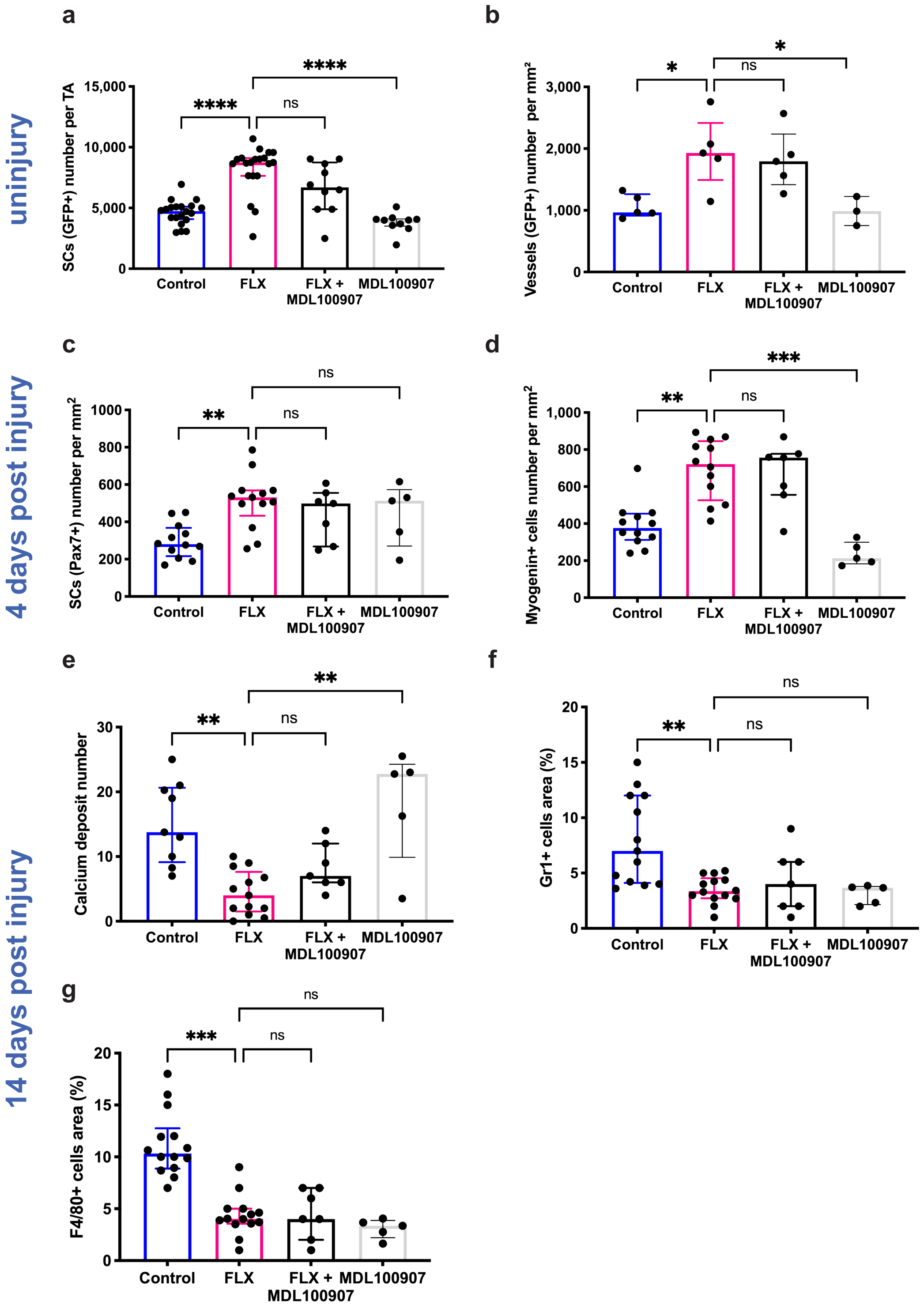

Supplementary Figure 5: Fluoxetine acts on muscle stem cells and muscle regeneration independently of the 5-HT2A receptor.

(a) Quantification of the SCs (GFP+) number per TA with cytometry analysis from *Tg:Pax7nGFP* mice in fluoxetine (FLX), FLX + MDL100907 (5-HT2A antagonist), MDL100907 and control group (n=20 control and FLX, 10 per other condition; 2 independent experiments). **(b)** Quantification of the vessels number by GFP immunostaining on TA sections from $\textit{Flk1}^{GFP/+}$ mice in FLX, FLX+ MDL100907, MDL100907 and control group (n=3 MDL100907, 5 per other condition). **(c)** Quantification of the SCs number by Pax7 immunostaining 4 days post injury on TA sections from control, FLX, MDL100907 and FLX+ MDL100907-treated C57Bl6 mice (n=13 control, 14 FLX, 7 FLX+ MDL100907, 5 MDL100907). **(d)** Quantification of the differentiating cells number by Myogenin immunostaining 4 days post injury on TA sections from control, FLX, MDL100907 and FLX+ MDL100907-treated C57Bl6 mice (n=14 control, 14 FLX, 7 FLX+ MDL100907, 5 MDL100907). **(e)** Quantification of the calcium deposit number stained with Hematoxylin and Eosin 14 days post injury on TA sections from control, FLX, MDL100907 and FLX+ MDL100907-treated C57Bl6 mice (n=10 control, 14 FLX, 7 FLX+ MDL100907, 5 MDL100907). **(f)** Percentage of Gr1+ (granulocytes) and **(g)** F4/80+ (macrophages) immune cells infiltration areas 14 days post injury on TA sections from control, FLX, MDL100907 and FLX+ MDL100907-treated C57Bl6 mice (n=14 control, 14 FLX, 7 FLX+ MDL100907, 5 MDL100907). The Kruskal-Wallis test for **(a)-(g).** All values are represented as median with interquartile range. * $p \le 0.05$, ** $p \le 0.01$, *** $p \le 0.001$, ****$p \le 0.0001$. Source data are provided as a Source Data file.

Supplementary Table 1: List of antibodies used for immunostainings.

Antigen (clone)	Host	Concentration	Reference
BrdU (Bu20a)	Mouse	1µg/ml	Dako M0744
CD31	Rat	15µg/ml	BD Pharmingen 550274
F4/80 (BM8)	Rat	0.5µg/ml	Thermo scientific MF-48000
GFP	Chicken	1µg/ml	Abcam ab13970
Laminin	Rabbit	0.69µg/ml	Sigma-Aldrich L9393
Ly-6C (Gr1)	Rat	0.5µg/ml	Caltag LabRM3030
Myogenin (F5D)	Mouse	4µg/ml	Thermo scientific MA5-11486
Pax7	Mouse	12µg/ml	DSHB
Pax7	Rabbit	6µg/ml	Abcam ab187339
MyHC type I (BA-D5)	Mouse	6µg/ml	DSHB
MyHC type IIA (BF-F3)	Mouse	6µg/ml	DSHB
MyHC type IIB (SC-71)	Mouse	6µg/ml	DSHB
Ki67	Rabbit	3µg/ml	Abcam ab15580
Secondary antibodies according to the primary Ab host	Donkey	0.5µg/ml	Jacksonimmuno<br>#711486152 (Rabbit)<br>#200162037 (Mouse)<br>#703546155 (Chicken)

Supplementary Table 2: List of antibodies used for western blots.

Antigen	Host	Concentration	Reference
5-HT1A	Rabbit	0.3µg/ml	Thermo scientific PA5-28090
5-HT1B	Rabbit	1µg/ml	Sigma SAB4501470
5-HT1D	Rabbit	1µg/ml	Invitrogen PA1-29462
5-HT1F	Rabbit	0.5µg/ml	Invitrogen PA5-51066
5-HT2A	Mouse	1µg/ml	Novus Biological NBP2-26091
5-HT2B	Rabbit	3µg/ml	Novus Biological NLS1187
5-HT2C	Rabbit	0.25µg/ml	Novus Biological NBP2-67100
SERT	Rabbit	4µg/ml	Alomone Labs AMT-004
GAPDH	Rabbit	1µg/ml	Cell Signaling 2118
HRP-linked secondary antibodies according to the primary Ab host	Goat<br>Horse	0.2µg/ml	Cell Signaling<br>#7074 (Rabbit)<br>#7076 (Mouse)

Supplementary Table 3: List of primers used for RT-qPCR.

Gene	Sequences
Pax7	Fwd: GCGAGAAGAAAGCCAAACAC<br>Rev: TCGGGTTCTGATTCCACATC
Htr1b	Fwd: CTCCATCTCTATTTCGTTGC<br>Rev: GTCTTGTTGGGTGTCTGTTT
Htr2a	Fwd: TGTGATGCTTTTAACATTGC<br>Rev: CCAACTTACTCCCATGCTAC
Sert	Fwd: TGACATCAGGAGGGGCGTAT<br>Rev: CAGCATTTCCTTCACGTCGC
Cyclin D1	Fwd: CCCTGACACCAATCTCCTCAAC<br>Rev: GCATGGATGGCACAATCTCCT
GAPDH	Fwd: TGATGACATCAAGAAGGTGGTGAAG<br>Rev: TCCTTGGAGGCCATGTAGGCCAT
