# Supplementary material for: Serotonin reuptake inhibitors improve muscle stem cell function and muscle regeneration in male mice
Source: Nat Commun. 2024 Jul 31;15:6457. doi: 10.1038/s41467-024-50220-4 (PMC11291725; doi:10.1038/s41467-024-50220-4)
Supplement: Supplementary file 3 — Reporting Summary [file 41467_2024_50220_MOESM3_ESM.pdf]

nature portfolio

Corresponding author(s): Fabrice CHRETIEN

Last updated by author(s): Apr 20, 2024

Reporting Summary

Nature Portfolio wishes to improve the reproducibility of the work that we publish. This form provides structure for consistency and transparency in reporting. For further information on Nature Portfolio policies, see our Editorial Policies and the Editorial Policy Checklist.

Statistics

For all statistical analyses, confirm that the following items are present in the figure legend, table legend, main text, or Methods section.

n/a	Confirmed	
☐	☒	The exact sample size (*n*) for each experimental group/condition, given as a discrete number and unit of measurement
☐	☒	A statement on whether measurements were taken from distinct samples or whether the same sample was measured repeatedly
☐	☒	The statistical test(s) used AND whether they are one- or two-sided<br>*Only common tests should be described solely by name; describe more complex techniques in the Methods section.*
☒	☐	A description of all covariates tested
☐	☒	A description of any assumptions or corrections, such as tests of normality and adjustment for multiple comparisons
☐	☒	A full description of the statistical parameters including central tendency (e.g. means) or other basic estimates (e.g. regression coefficient) AND variation (e.g. standard deviation) or associated estimates of uncertainty (e.g. confidence intervals)
☒	☐	For null hypothesis testing, the test statistic (e.g. *F*, *t*, *r*) with confidence intervals, effect sizes, degrees of freedom and *P* value noted<br>*Give P values as exact values whenever suitable.*
☒	☐	For Bayesian analysis, information on the choice of priors and Markov chain Monte Carlo settings
☒	☐	For hierarchical and complex designs, identification of the appropriate level for tests and full reporting of outcomes
☒	☐	Estimates of effect sizes (e.g. Cohen's *d*, Pearson's *r*), indicating how they were calculated

Our web collection on statistics for biologists contains articles on many of the points above.

Software and code

Policy information about availability of computer code

Data collection	All data generated or analyzed during this study are included in this article and its supplementary information files.
Data analysis	All the software used for data analysis is described in the Methods section: GraphPad Prism software version 9, StepOne Plus RT PCR software v2.1, Columbus Conductor™ software (Perkin Elmer Technologies), high-content analysis, MuscleJ 70, in ImageJ software environment, Summit v4.3 software from DakoCytomation and FloJo software, BD FACSDIVA software (BD Biosciences),Chart v4.2.3 (PowerLab 4/25 ADInstrument, PHYMEP France), ImageJ software

For manuscripts utilizing custom algorithms or software that are central to the research but not yet described in published literature, software must be made available to editors and reviewers. We strongly encourage code deposition in a community repository (e.g. GitHub). See the Nature Portfolio guidelines for submitting code & software for further information.

Data

Policy information about availability of data

All manuscripts must include a data availability statement. This statement should provide the following information, where applicable:

- Accession codes, unique identifiers, or web links for publicly available datasets
- A description of any restrictions on data availability
- For clinical datasets or third party data, please ensure that the statement adheres to our policy

All data generated or analyzed during this study are included in this article and its supplementary information files. The uncropped gel or blot figures and original data underlying Figures 1-4 and Supplementary Figures 1-5 are provided in a source data file.

Research involving human participants, their data, or biological material

Policy information about studies with human participants or human data. See also policy information about sex, gender (identity/presentation), and sexual orientation and race, ethnicity and racism.

Reporting on sex and gender	n/a
Reporting on race, ethnicity, or other socially relevant groupings	n/a
Population characteristics	n/a
Recruitment	n/a
Ethics oversight	n/a

Note that full information on the approval of the study protocol must also be provided in the manuscript.

Field-specific reporting

Please select the one below that is the best fit for your research. If you are not sure, read the appropriate sections before making your selection.

☒ Life sciences ☐ Behavioural & social sciences ☐ Ecological, evolutionary & environmental sciences

For a reference copy of the document with all sections, see nature.com/documents/nr-reporting-summary-flat.pdf

Life sciences study design

All studies must disclose on these points even when the disclosure is negative.

Sample size	This present work based on animal models did not require a sample size calculation. We have specified the n values of each group for each experiment. No prior calculation was made for the sample size calculation. In order to demonstrate a significant difference using non-parametric tests on animal models, we considered that the n value should be at least 3 and ideally greater than or equal to 5 for the control groups, and the n value should be at least 5 and ideally greater than or equal to 7 for a treatment group, with ideally at least one replication on two independent experiments. In order to demonstrate a significant difference using non-parametric tests on cellular experiments, we considered that the n value had to be a minimum of 30 cells per group on rare populations (for example, cells cultured on single fibers) and the n value had to be greater than or equal to 100 for other cell types.
Data exclusions	There were no prior exclusion criteria and no data were excluded
Replication	Most of the in vivo experiments were replicated on two independent sets except for the manipulations involving rare transgenic mice (Most of the in vivo experiments were replicated on two independent sets except for the manipulations involving rare transgenic mice (one independent experiment was performed with the TPH1-/- mice strain and with the Pax7-CreERT(2): :tetO1B mice strain). In vitro, experiments on immortalized cells were repeated three times. Except for the initial results of the Luminex intramuscular cytokine assays (data removed from the revised article due to lack of robustness), all replicates were consistent.
Randomization	The animals used in this study were inbred, same strain, same sex and same age. All animals were simultaneously randomized to the different control or treatment groups without considering any other variable.
Blinding	During the period of treatment delivery, no blinding was implemented, inbred mice of the same sex and age being strictly comparable. After killing the animals, the different samples were marked with the same number per mouse. The manual analyzes were thus carried out blindly, the only information available per sample being the number previously assigned. Unblinding of the analysis was carried out during the statistical analysis. Certain analyzes were automated without the need for blinding (see Fiji, MuscleJ, Columbus).

Reporting for specific materials, systems and methods

We require information from authors about some types of materials, experimental systems and methods used in many studies. Here, indicate whether each material, system or method listed is relevant to your study. If you are not sure if a list item applies to your research, read the appropriate section before selecting a response.

Materials & experimental systems

n/a	Involved in the study	
☐	☒	Antibodies
☐	☒	Eukaryotic cell lines
☒	☐	Palaeontology and archaeology
☐	☒	Animals and other organisms
☒	☐	Clinical data
☒	☐	Dual use research of concern
☒	☐	Plants

Methods

n/a	Involved in the study	
☒	☐	ChIP-seq
☐	☒	Flow cytometry
☒	☐	MRI-based neuroimaging

Antibodies

Antibodies used

5-HT1A antibody: lotTI2641019D, Thermo scientific #PA5-28090
5-HT1B antibody: lot 3112009, Sigma #SAB4501470
5-HT1D antibody: lot 012845448F, Invitrogen #PA1-29462
5-HT1F antibody: lot 012845071, Invitrogen #PA5-51066
5-HT2A antibody: lot P1 E101218, Novus Biological #NBP2-26091
5-HT2B antibody: lot 42088, Novus Biological #NLS1187
5-HT2C antibody: lot HK0703, Novus Biological #NBP2-67100
SERT antibody: lot AMT004AN0502, Alomone Labs #AMT-004
GAPDH antibody: lot 14, Cell Signaling #2118
HRP-linked secondary antibodies according to the primary Ab host: Cell Signaling, #7074 (rabbit), #7076 (mouse)
BrdU antibody (clone: Bu20a): Dako #M0744
CD31 antibody: lot 9002678, BD Pharmingen #550274
F4/80 antibody (clone BM8): lot 1389811A, Thermo scientific #MF-48000
GFP antibody: lot GR89472-22, Abcam #ab13970
Laminin antibody: lot 0000128425, Sigma-Aldrich #L9393
Ly-6C antibody (clone Gr1): lot MA1-81899, Caltag LabRM3030
Myogenin antibody (clone F5D): lot 2407527, Thermo scientific #MA5-11486
Pax7 antibody: DSHB
Pax7 antibody, Abcam ab187339
Ki67 antibody, Abcam ab15580
MyHC type I antibody (clone BA-D5): DSHB
MyHC type IIA antibody (clone BF-F3): DSHB
MyHC type IIB antibody (clone SC-71): DSHB
Secondary antibodies according to the primary Ab host: Jacksonimmuno #711486152 (Rabbit), #200162037 (Mouse), #703546155 (Chicken)
All antibodies used was provided in supplementary table 1 and 2.

Validation

For each primary antibody, we carried out validations with positive and/or negative controls according to the information available on the manufacturer's website.
5-HT1A antibody: lotTI2641019D, Thermo scientific #PA5-28090: positive control used: mouse brain sample, negative control used: mouse kidney sample
5-HT1B antibody: lot 3112009, Sigma #SAB4501470, positive control used: mouse brain sample, negative control used: mouse liver sample
5-HT1D antibody: lot 012845448F, Invitrogen #PA1-29462, positive control used: mouse brain sample
5-HT1F antibody: lot 012845071, Invitrogen #PA5-51066, positive control used: mouse brain sample, negative control used: mouse liver sample
5-HT2A antibody: lot P1 E101218, Novus Biological #NBP2-26091, positive control used: mouse brain sample
5-HT2B antibody: lot 42088, Novus Biological #NLS1187, positive control used: mouse heart and brain sample
5-HT2C antibody: lot HK0703, Novus Biological #NBP2-67100: positive control used: mouse brain sample
SERT antibody: lot AMT004AN0502, Alomone Labs #AMT-004: positive control used: mouse brain sample
GAPDH antibody: lot 14, Cell Signaling #2118: positive control used: mouse brain sample
BrdU antibody (clone: Bu20a): Dako #M0744, negative control used: C2C12 sample
CD31 antibody: lot 9002678, BD Pharmingen #550274: positive control used: large vessels, negative control used: C2C12 sample
F4/80 antibody (clone BM8): lot 1389811A, Thermo scientific #MF-48000, positive control used: mouse spleen sample, negative control used: C2C12 sample
GFP antibody: lot GR89472-22, Abcam #ab13970, negative control used: C2C12 sample
Laminin antibody: lot 0000128425, Sigma-Aldrich #L9393: positive control used: mouse kidney sample, negative control used: C2C12 sample
Ly-6C antibody (clone Gr1): lot MA1-81899, Caltag LabRM3030: positive control used: mouse bone marrow sample, negative control used: C2C12 sample

Myogenin antibody (clone F5D): lot 2407527, Thermo scientific #MA5-11486: positive control used: C2C12 sample, negative control used: mouse brain sample
Pax7 antibody: DSHB: positive control used: C2C12 sample, negative control used: mouse brain sample
Pax7 antibody, Abcam ab187339: positive control used: C2C12 sample, negative control used: mouse brain sample
Ki67 antibody, Abcam ab15580: positive control used: C2C12 sample, negative control used: mouse brain sample
MyHC type I antibody (clone BA-D5): DSHB: positive control used: mouse muscle sample, negative control used: mouse brain sample
MyHC type IIA antibody (clone BF-F3): DSHB: positive control used: mouse muscle sample, negative control used: mouse brain sample
MyHC type IIB antibody (clone SC-71): DSHB: positive control used: mouse muscle sample, negative control used: mouse brain sample
For each secondary antibody, at the time of use, a negative control was used (no primary antibody and exposure to secondary antibody only).
Secondary antibodies according to the primary Ab host: Jacksonimmuno #711486152 (Rabbit), #200162037 (Mouse), #703546155 (Chicken)
HRP-linked secondary antibodies according to the primary Ab host: Cell Signaling, #7074 (rabbit), #7076 (mouse)

Eukaryotic cell lines

Policy information about cell lines and Sex and Gender in Research

Cell line source(s)	C2C12 (CRL-1772, from ATCC) is a immortalized myoblast cell line from male Mus Musculus mouse<br>Primary muscle stem cell was sorted by FACS from male Mus Musculus Tg:Pax7nGFP mouse
Authentication	no authentication procedure used
Mycoplasma contamination	cell lines were not tested for mycoplasma contamination
Commonly misidentified lines (See ICLAC register)	no misidentified lines used

Animals and other research organisms

Policy information about studies involving animals; ARRIVE guidelines recommended for reporting animal research, and Sex and Gender in Research

Laboratory animals	6 weeks old male mice were used in this study and different strains were used: the wild-type C57Bl/6RJ, Tg:Pax7nGFP, Flk1GFP/+ , TPH1-/-, Pax7-CreER(T2) and tetO1B. All the genotyping were performed by standard PCR methods. Mice were housed on a 12:12 light/dark cycle in a pathogen free facility with controlled temperature and humidity. Food and drink were given ad libitum.
Wild animals	Wild inbred 6-week-old male C57Bl6 animals were ordered from Charles River Company and transported by adapted package to the laboratory animal facility. Mice were housed on a 12:12 light/dark cycle in a pathogen free facility with controlled temperature and humidity. Food and drink were given ad libitum. After in vivo experiment, all mice were killed by cervical dislocation to collect the different samples
Reporting on sex	In this study, only male mice were used to limit the known influence of sex hormones on the muscle phenotype
Field-collected samples	Mice were housed on a 12:12 light/dark cycle in a pathogen free facility with controlled temperature and humidity. Food and drink were given ad libitum. After in vivo experiment, all mice were killed by cervical dislocation to collect the different samples. No filed collected samples are used in the study.
Ethics oversight	All procedures in this study were approved by the Animal Care and Use committee at the Institut Pasteur (CETEA 2014-0040). For the in situ experiments, all procedures were conducted in conformity with European rules for animal experimentation (French Ethical Committee APAFIS#33392-2021100416152925, November 19, 2021).

Note that full information on the approval of the study protocol must also be provided in the manuscript.

Flow Cytometry

Plots

Confirm that:

- [x] The axis labels state the marker and fluorochrome used (e.g. CD4-FITC).
- [x] The axis scales are clearly visible. Include numbers along axes only for bottom left plot of group (a 'group' is an analysis of identical markers).
- [x] All plots are contour plots with outliers or pseudocolor plots.
- [x] A numerical value for number of cells or percentage (with statistics) is provided.

Methodology

Sample preparation	All the muscles of the hind limbs from Tg:Pax7nGFP mice were dissected in cold DMEM-Glutamax. Muscles were then

	chopped with small scissors and put in a 50 ml Falcon tube with collagenase 0.08% (Sigma-Aldrich, #C51385), trypsin 0.08% (Sigma-Aldrich, #G6452) and DNAse 1UI/mL (Roche, #04716728001) at 37°C with gentle agitation. After 20 minutes, the supernatant was collected in 20% serum placed on ice, and the collagenase/trypsin solution was added to continue the digestion. Once muscle is completely digested, the solution was filtrated using 40µm cell strainers and analyzed by FACS. Cells were labelled with propidium Iodide 10 µg/ml (Sigma-Aldrich#P4170) to exclude dead cells and displayed using the PE (Phycoerythrin, Red) channel on the FACS profile.
Instrument	FACS analysis was done using a FACSaria (Beckman). Cell sorting was done with Aria III (BD Biosciences).
Software	All analyses and quantitation were performed using Summit v4.3 software from DakoCytomation and FloJo software. Cell sorting was done with BD FACSDIVA software (BD Biosciences).
Cell population abundance	The target subpopulation of satellite cells sorted with endogenous GFP from Tg:Pax7nGFP mice corresponds to about 3% of the total population with a sorting purity already demonstrated > 98% (reference 15)
Gating strategy	A subset of the cells were selected on the FSC-A medium size and SSC-A low granularity gates. Finally, the cells of interest were selected on the GFP vs PE gates as GFP+-PE- cells.

☒ Tick this box to confirm that a figure exemplifying the gating strategy is provided in the Supplementary Information.
